# Ripening and Storage Time Effects on the Aromatic Profile of New Table Grape Cultivars in Chile

**DOI:** 10.3390/molecules25245790

**Published:** 2020-12-08

**Authors:** Cristina Ubeda, Mariona Gil i Cortiella, Luis Villalobos-González, Camila Gómez, Claudio Pastenes, Álvaro Peña-Neira

**Affiliations:** 1 Departamento de Nutrición y Bromatología, Toxicología y Medicina Legal, Facultad de Farmacia, Universidad de Sevilla, C/Profesor García González 2, 41012 Sevilla, Spain; c_ubeda@us.es; 2 Facultad de Ciencias, Instituto de Ciencias Biomédicas, Universidad Autónoma de Chile, 8910060 Santiago, Chile; 3 Facultad de Ingeniería, Instituto de Ciencias Químicas Aplicadas, Universidad Autónoma de Chile, 8910060 Santiago, Chile; Mariona.gil@uautonoma.cl; 4 Departamento de Producción Agrícola, Facultad de Ciencias Agronómicas, Universidad de Chile, Santa Rosa 11314, La Pintana, 8820000 Santiago, Chile; luisvillalobosg1@gmail.com (L.V.-G.); cpastene@uchile.cl (C.P.); 5 Department of Agro-Industry and Enology, Faculty of Agronomical Sciences, University of Chile, Santa Rosa 11315, La Pintana, 8820000 Santiago, Chile; camila.gomez.c@gmail.com

**Keywords:** table grape, ripening, storage time, volatile compounds, aroma

## Abstract

The aim of this study was to determine the volatile profiles of new seedless table grape cultivars Timco™, Magenta™, Krissy™ and Arra15™ and compare them with the traditional table grape variety Crimson seedless. The volatile profiles were extracted employing solid-phase microextraction and analyzed with gas chromatography coupled with mass spectrometry. Terpenes were present in very different proportions, with the Magenta, Krissy, and Arra15 varieties showing much higher quantities than Crimson and Timco. β-Ionone and octanal, important indicators in the aromatic flavor quality of table grapes, were present in higher levels in Crimson and Arra15, and this might be responsible for driving consumer preference. These compounds significantly increased during ripening, except in Crimson, and gradually decreased from harvest to the end of the storage in all the cultivars. Evolution during ripening was different depending on the variety but the general tendency terpenes was to increase from veraison to harvest. A postharvest study revealed that Crimson could have a better conservation of the volatile components during postharvest storage compared with Timco and Krissy. These results could help in plant breeding programs and to make decisions for new planting according to needs for storing fresh table grapes given distances to consumer markets.

## 1. Introduction

Grapes are one of the main crops in the world, with an annual production of over 77.8 million tons, of which approximately 36% are used for fresh consumption [1]. Table grape consumption is increasing all around the world; hence, new table grape varieties are being derived from breeding programs in different countries. These breeding programs are shaped according to the demands of consumers [2]. While fruit breeding programs have many different goals, including resistance to abiotic and biotic stresses, tree architecture, precocity, and productivity, they all have a common need to develop fruits with a wide spectrum of sizes, colors, firmness levels, textures, aromas, and flavors [3]. Especially in recent years, the demand has increased for cultivars of table grapes with better taste, and aromatic compounds are an important part of the quality of these cultivars [4,5]. Grapes are non-climacteric fruits [6], and berry development follows a double-sigmoidal curve, with two phases of growth separated by a lag phase [7]. The transition between the growing phases is marked by the onset of ripening, which is called veraison, which triggers physiological changes until maturity is reached through berry softening, sugar accumulation, water influx, lowered acidity and astringency, and changes in skin color in red varieties [8], as well as the synthesis of volatile compounds and/or precursors that influence and determine the varietal aromatic characteristics. These aromas are related to the grape variety including monoterpenes and sesquiterpenes, C13-norisoprenoids, volatile sulfur compounds, and methoxypyrazines [9]. There is no standard agreement between growers and consumers about the optimal time at which to harvest table grapes [10].

Although the balance between sweetness and acidity is a basic concept in one’s judgment of the quality of many fruits as table grapes, to improve the taste, it is also necessary to know the composition, concentrations, and evolution of volatile compounds in fruit in the field during ripening and storage after harvesting [10,11].

The land area used for table grapes in Chile during the season of 2019/2020 was 47,834 ha, with a production of 835,000 [12]. According to United States Department of Agriculture (USDA) estimates, Chile exports approximately 80% of all table grape production as the main table grape exporter in the Southern Hemisphere and the world. Grape exports for the 2019/2020 season reached approximately 657,000 tons [12]. While known for such popular grape varieties as Red Globe, Crimson, and Thompson Seedless, Chile has been introducing numerous new varieties from several national and international breeding programs to the world market [13,14], with a focus on superior size, condition, and taste. In the last season, 83% of the volume corresponded to traditional varieties (Red Globe, Crimson, Thompson, Flame, Sugraone, and Autumn Royal), and the remaining 17% accounted for over 30 licensed new varieties, such as Timco™, Sweet Celebration™, Arra15™, Allison™, Magenta™, Scarlotta™, Pristine™, Sable™, Krissy™, and Maylen [15]. Considering that there is a lack of information about the aromatic compositions of some of these new varieties during ripening and postharvest storage, the aim of this work was to study the aromatic compositions of the new red varieties, Magenta™, Krissy™, and Timco™, and a new white variety, Arra-15™, compared with the second most planted red traditional table grape variety in Chile, Crimson seedless.

## 2. Results and Discussion

### 2.1. Progression of Basic Physical and Chemical Variables in the Table Grape Berries

**Figure 1:**
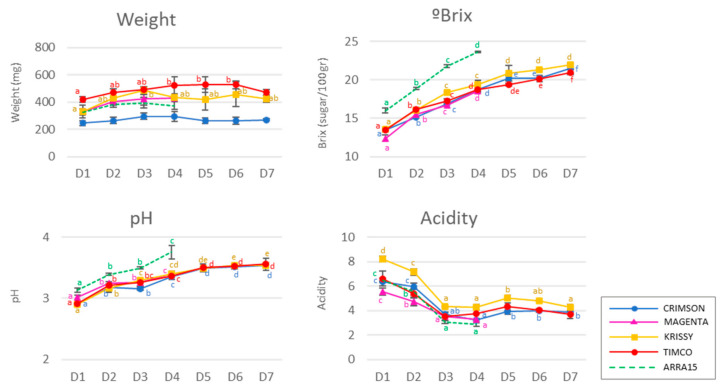
Berry general analyses: weight, ° Brix, pH, and acidity. Different superscript letter indicates statistically significant differences among the sampling times D1–D7 (*p* < 0.05). Sampling along harvest: **D1**, veraison; **D2**, 12 DAV (days after veraison); **D3**, 26 DAV; **D4**, 37 DAV. Sampling along storage: **D5**, 54 DOS (days of storage); **D6**, 75 DOS; **D7**, 108 DOS.

Typical chemical variables are shown in Figure 1. The soluble solids among red and white cultivars were different on the different sampling dates due to their distinct precocities and ripening behaviors. The red cultivars ranged from 12.4° to 13.6° Brix in the first stage (D1) and increased until harvest (D4) to 18.7°–19.4° Brix. In the case of the white variety, the concentration of soluble solids was 16.0° ± 0.3° Brix at D1, increasing to 23.7° ± 0.1° Brix at D4. The red varieties studied during postharvest reached 19.4°–22.0° Brix. On average, the berry weight increased to different extents during ripening in all the grapes studied. At commercial maturity, the berries weighed between 294 and 522 mg, which corresponded to the Crimson and Timco varieties, respectively, with their weights decreasing during storage, except for the Krissy variety (Figure 1). Additionally, as expected, the pH increased, and the acidity decreased significantly during ripening for all the varieties. During storage, these two parameters remained nearly constant. The white variety, Arra15, despite having an intermediate weight among all the varieties, reached the highest soluble solid concentration and exhibited a higher pH from D1 until harvest at D4.

### 2.2. Comparison among the Different Table Grape Volatile Profiles

Forty-one volatile compounds were determined in the five grape varieties analyzed. Among them, the terpene chemical group was the most abundant (qualitatively), accounting for 14 compounds, followed by aldehydes (13), alcohols (9), ketones (3), C13-norisoprenoids (1), and acids (1) (Table 1). Due to their distinctive characteristics, the general parameters of each grape variety were different at the commercial maturity stage (D4, defined by the producer in relation to the soluble solids of the berries) (Table 1; Figure S1, Supplementary Materials).

**Figure 2:**
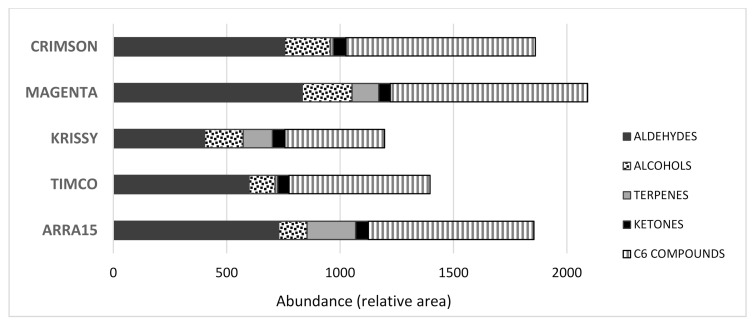
Total amounts of volatile compounds found in the table grapes analyzed.

As mentioned above, when harvested for commercialization, the grape soluble solids are usually lower in red varieties than in white ones. At this point, their volatile profiles were quite different, as can be observed in Figure 2.

The Magenta variety accounted for the highest total concentration of volatile compounds, closely followed by Arra15, in contrast to Krissy, which was the grape variety with the lowest total quantity of volatile molecules. Additionally, the presence of the different main chemical families of volatile compounds was different depending on the variety. Among them, terpenes were very different in proportion, with the Magenta, Krissy, and Arra15 varieties showing much higher quantities of terpenes than Crimson and Timco grapes. It is important to consider that terpenes have a low perception threshold and usually contribute to the citrus/green/fresh aroma of a fruit [16,17], making them probably responsible for the Magenta, Krissy, and Arra15 perceived aromas. Apart from these general differences, there were observed individual characteristics. Not all the volatile compounds present in the grapes contribute to the same extent to the aroma of the fruit. In fact, some of them are not odoriferous [18]. In this sense, a recent study carried out a large screening of the aromatic profiles of 20 table grape cultivars, determining that C6, terpene, and C13-norisoprenoid compounds were the main contributors to the aromas of the table grapes analyzed [5]. In this study, the C6 compounds found were aldehydes and alcohols, including hexenal, (*E*)-2-hexenal, 2,4-hexadienal, hexanol, (*Z*)-3-hexenol, (*E*)-2-hexenol, and 2-ethyl-1-hexanol (Table 1, Figure 2). This group of molecules is well known and contributes to the green nuances of grapes, being products of the enzymatic breakdown of unsaturated fatty acids [19]. They are derived from linoleic and linolenic acid and have been described as the basic background volatiles in table grape berries [5,20]. Among them, it was found that hexanal and (*E*)-2-hexenal were the major compounds in all the grape varieties, which was in agreement with previous studies [11,21,22]. The amount of hexanal found in Krissy was significantly lower than the quantities present in the other four varieties analyzed. The ratio of hexanal to (*E*)-2-hexenal was recently correlated with sweetness [22], and these compounds have also been quoted as the most active C6 compounds in table grapes and, therefore, are important contributors to the perceived aroma of this fruit [5]. 

With respect to the alcohols, the compositions among the grape varieties analyzed were significantly different in most of the cases. Thus, C6-alcohols, which are contributive volatile compounds to the aroma of table grapes [5], hexanol, and (*Z*)-3-hexenol were present in Crimson grapes in significantly higher quantities compared to the other varieties, and the same was true of (*E*)-2-hexenol in the Arra15 white variety (Table 1). These compounds provide green grass/herbaceous aromas to the fruits [23]. Hexanol was the major volatile alcohol in all the varieties, followed by 2-ethyl-1-hexanol, except for Krissy, in which the major alcohol was pentanol. In comparing the total amount of C6 compounds, aldehydes and alcohols, it was observed that the Magenta variety reached the highest quantity on average (870), followed by the Crimson (836), Arra15 (730), Timco (621), and Krissy (440) varieties (Figure 2). These results could be reflected in the stronger green sensory character of the Crimson, Magenta, and Arra15 varieties compared to the other grapes.

In addition to C6 compounds, another essential contributor to grape aromas is the terpene group, which provides positive characteristic floral/fruity/citric aromas [22,24]. As mentioned above, two clear groups could be formed on the basis of the terpene compositions, with Magenta, Krissy, and Arra15 showing higher abundances. The differences in the grapes at commercial maturity were remarkable for some terpenes, including β-cymene, linalool, (*E*)-citral, nerol, and geraniol. Among them, geraniol was found to be the most abundant terpene in all the varieties except Crimson grapes, in which case the most abundant was limonene, followed by geraniol (Table 1). Geraniol is one of the main compounds contributing to the distinctive floral characteristics of Muscat varieties [25]. The dominance of geraniol in table grapes was previously described [26], but this result is in contrast with the findings of other authors, who described linalool as the major terpene, followed by geraniol [21,27]. This divergence may be because, unlike wine grapes, which show abundant free monoterpenes in the pulp, table grapes are rich in free monoterpenes in the skin [5], and, in this study, the whole berry was crushed but the volatile compounds in the skin were not analyzed separately. In the terpenic varieties, following geraniol, nerol was the most abundant terpene present in the berry, and, in the Crimson and Timco varieties, limonene was the other most abundant terpene (Table 1).

It is difficult to establish the compounds responsible for the food preferences of consumers. However, in the study by Wu et al. previously mentioned [5], β-ionone and octanal were highlighted as important indicators for evaluating or determining the aromatic flavor quality of table grapes. These compounds were present in higher amounts in Crimson and Arra15 at the commercial ripening stage and might be responsible for driving consumer preference (Table 1).

### 2.3. Evolution of Volatile Compounds during Ripening

**Figure 3:**
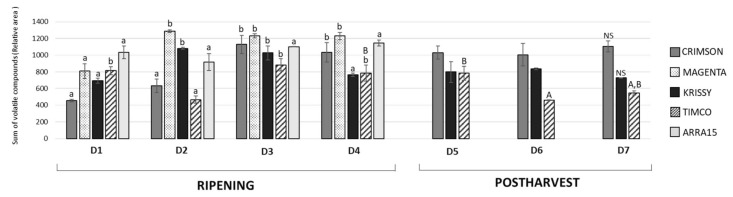
Evolution of the total volatile amounts during ripening and storage of the grapes (expressed in relative area). Values in the bars with different superscript letters indicate statistically significant differences (*p* < 0.05). Lower case (a): ANOVA test applied among ripening samples (D1–D4); Upper case (A): ANOVA test applied among harvest and storage samples (D4–D7). ns: nonsignificant differences found. Sampling along harvest: **D1**, veraison; **D2**, 12 DAV (days after veraison); **D3**, 26 DAV; **D4**, 37 DAV. Sampling along storage: **D5**, 54 DOS (days of storage); **D6**, 75 DOS; **D7**, 108 DOS.

Grapes are a non-climacteric fruit; therefore, from harvest, berries do not ripen further. Since the aroma is one of the most important attributes driving consumer preference, it is necessary to ensure that grapes have reached the ideal concentration of the volatiles associated with positive aromas at the time of harvest. Excluding ethanol and ethyl acetate, which are associated with the natural progression of the ripening of the fruit and over-maturity, *Vitis vinifera* berry volatile compounds are mainly formed through (a) fatty-acid metabolism of C18 linoleic and linolenic acids, leading to C6–C13 volatile compounds, (b) amino-acid metabolism, giving rise to volatile compounds such as 2-phenylethanol and benzyl alcohol derived from the metabolism of aromatic and branch-chained amino acids, and (c) terpene biosynthetic pathways, leading to the formation of cyclic and oxygenated monoterpenes, sesquiterpenes, and apocarotenoids [28]. Figure 3 shows the progression of the complete volatile fraction from veraison through ripening, harvest, and the storage period. 

**Figure 4:**
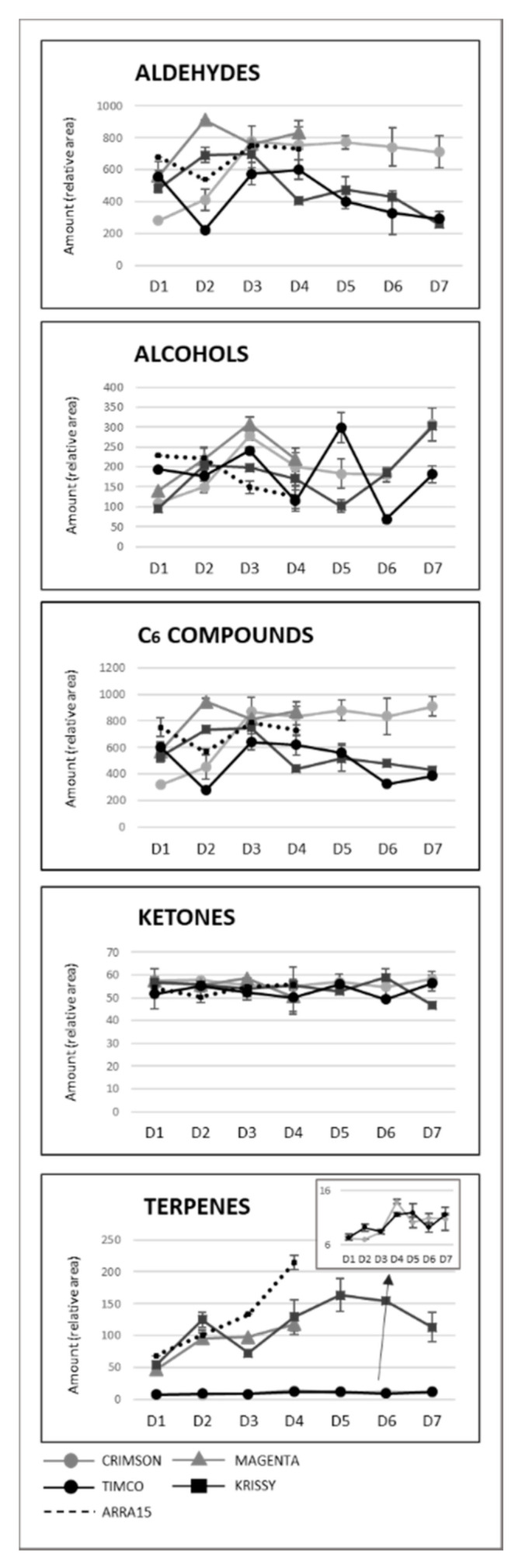
Evolution of the main chemical groups of volatile compounds during ripening and storage. Sampling along harvest: **D1**, veraison; **D2**, 12 DAV (days after veraison); **D3**, 26 DAV; **D4**, 37 DAV. Sampling along storage: **D5**, 54 DOS (days of storage); **D6**, 75 DOS; **D7**, 108 DOS.

As can be observed, Magenta and Krissy reached their maximum volatile concentrations at D2 (12 DAV), followed by Crimson and Timco at D3 (26 DAV), and the white variety Arra15 at the harvesting date (37 DAV). At D3 (26 DAV), the formation of volatile compounds peaked, with no further changes to D4 (37 DAV), except for Krissy, which showed a significantly decreased total amount of volatile compounds. Indeed, Krissy was the variety with the lowest quantity of these secondary metabolites. Interestingly, the white variety did not show significant variation in the total amount of volatiles during ripening. With respect to C6 compounds, the progression pattern was strongly dependent on the grape cultivar. Thus, in Crimson, Magenta, and Krissy cultivars, most of these aldehydes and alcohols increased during ripening until harvest, as observed from Figure 4, in which the total aldehyde, alcohol, C6-compound, ketone, and terpene evolutions are represented, with the detailed evolution for compounds shown in Table S1 (Supplementary Materials).

**Figure 5:**
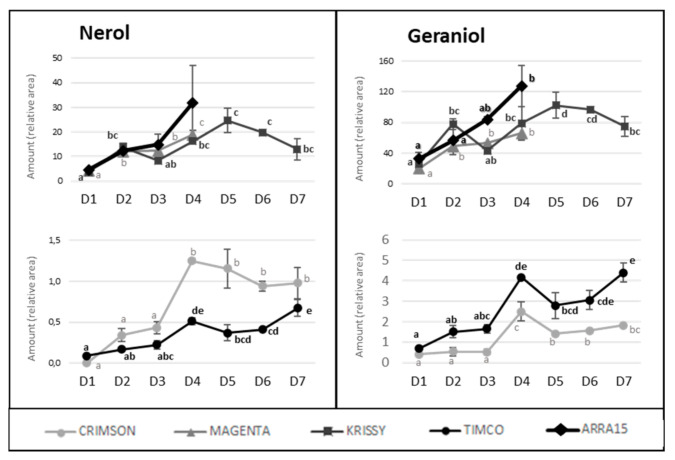
Progression of nerol and geraniol amounts along maturity and storage in all the table grapes. Different superscript letter indicates statistically significant differences among the sampling times D1–D7 (*p* < 0.05). Sampling along harvest: **D1**, veraison; **D2**, 12 DAV (days after veraison); **D3**, 26 DAV; **D4**, 37 DAV. Sampling along storage: **D5**, 54 DOS (days of storage); **D6**, 75 DOS; **D7**, 108 DOS.

In contrast to these cultivars, in the cases of Timco and Arra15, just half of the C6 compounds increased from D1 (veraison) to D4 (37 DAV). Among all the aldehydes and alcohols determined in this study, heptanal, (*E*,*E*)-2,4-heptadienal, (*E*)-2-octenal, 2-methyl-6-heptenol, 2-ethyl-1-hexanol, benzyl alcohol, and 2-phenylethanol were the compounds that increased in all the grape cultivars from veraison to harvest. As mentioned above, octanal is a volatile compound whose concentration has been related to acceptance by consumers. In all cases, this compound significantly increased its presence in berries during ripening, except in Crimson, which did not present significant differences between D1 and D4. Contrasting these results for C6 compounds, the evolution of terpenoid amounts during the ripening stages seemed to follow a general pattern between varieties. Nerol and geraniol were the terpenes that increased the most during maturation of the grapes (Figure 5). Hence, the general tendency among the 14 terpenes determined was to increase from veraison to the harvest date (Figure 4 and Figure 5), not including eucalyptol, β-cymene, and terpinen-4-ol. This result agrees with previous reports on table grapes [11,29].

**Figure 6:**
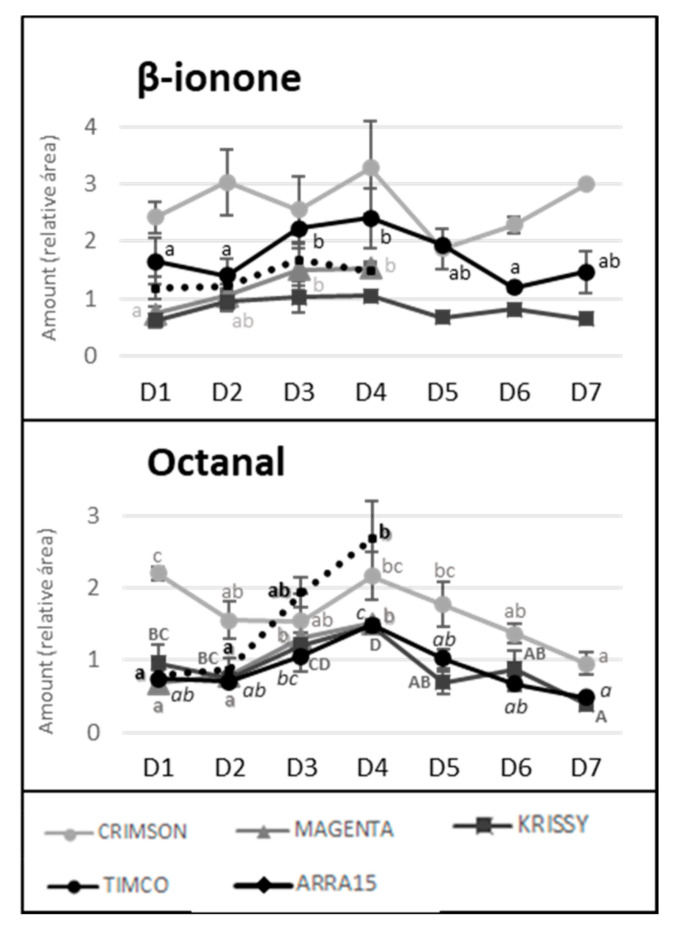
Evolution of β-ionone and octanal quantities along maturity and storage in all the table grapes. Different superscript letter indicates statistically significant differences among the sampling times D1–D7 (*p* < 0.05). Sampling along harvest: **D1**, veraison; **D2**, 12 DAV (days after veraison); **D3**, 26 DAV; **D4**, 37 DAV. Sampling along storage: **D5**, 54 DOS (days of storage); **D6**, 75 DOS; **D7**, 108 DOS.

Despite the general tendency of the increment of terpenoids between D1 and D4, several of these molecules reached their maximum values before the highest sugar level was reached (D4). Figure 4 reflects the evolution of terpene compounds maximizing the evolution of Crimson and Timco to clearly appreciate the changes along the periods studied. Considering the red varieties analyzed, (*Z*)-citral, (*E*)-citral, nerol, and geraniol reached their maximum values at the harvest date and, conversely, citronellol, *p*-cymene, γ-terpinene, and terpinen-4-ol accounted for the highest quantity increasing rates during the early stages of ripening (D1: veraison and D2: 12 DAV) (Figure 5). This might be related to the continuous formation of glycosides during the maturation period. These molecules are secondary metabolites of the plants, consisting of a non-sugar component, called aglycone, attached to one or more sugars [30]. Due to their nonpolar or semipolar nature, glycosylation increases their water solubility, allowing the transportation, accumulation/storage, and detoxification of these compounds [30]. Terpenoids are commonly found in berries in their glycoside form. In fact, glycosylated forms are frequently more common than free terpenes [17]. Enzymes (glycoside transferases and hydrolases) involved in glycosylation are synthesized from veraison in the grape berries [31]. Consequently, possibly depending on the characteristics of each terpene, the maximum quantity of their free form is reached in the early stages of maturation and, afterward, the berry tends to glycosylate these compounds in greater quantity, reflecting them as a diminution or a stabilization of the quantity in D3 (26 DAV) and D4 (37 DAV). Apart from terpenoids, another remarkable isoprenoid evolution was found for β-ionone. As mentioned before, this C13-norisoprenoid with violet/wood/raspberry nuances seems to be of strong importance to table grape consumer preferences [5]. In all the grape cultivars, β-ionone increased from veraison to harvest, ranging maximum levels at D3 (26 DAV) or D4 (37 DAV), depending on the variety (Figure 6). This could be an important point to consider in the decision of the harvesting date. Considering its important role in consumer acceptability, the fluctuation of β-ionone during maturity would be of special interest to the producer.

### 2.4. Effects on Volatile Compounds during Storage Time

**Figure 7:**
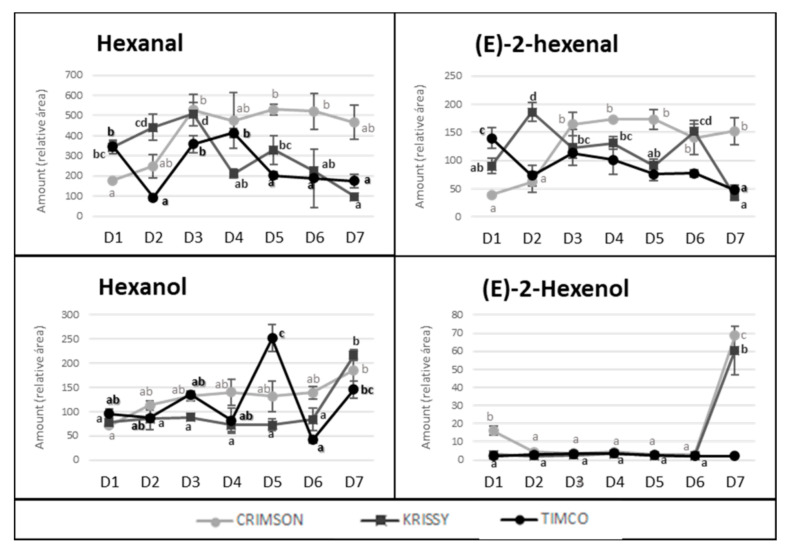
Evolution of hexanal, hexanol, (*E*)-2-hexenal, and (*E*)-2-hexenol amounts along maturity and storage of Crimson, Krissy, and Timco varieties. Different superscript letter indicates statistically significant differences among the sampling times D1–D7 (*p* < 0.05). Sampling along harvest: **D1**, veraison; **D2**, 12 DAV (days after veraison); **D3**, 26 DAV; **D4**, 37 DAV. Sampling along storage: **D5**, 54 DOS (days of storage); **D6**, 75 DOS; **D7**, 108 DOS.

The storage time is a crucial point in the production and commercialization of every fruit because, currently, food spends an extensive time at this stage due, among other reasons, to the globalized market and long distances between production places and selling locations and to constant production management over time. Very little is known about the behavior of the volatile fraction of table grapes during fresh storage, with studies having mainly focused on terpenoids [27,32,33]. In the present study, the total amount of volatile compounds in Crimson and Krissy cultivars did not vary significantly during the postharvest time (Figure 3). In contrast, the amount in Timco decreased from D5 (54 DOS) to D6 (75 DOS) and remained constant at D7 (108 DOS), indicating that Crimson and Krissy could have a better conservation of the volatile fraction postharvest compared to Timco. In analyzing results more deeply, the storage time had an important effect on the aldehydes from the harvest to the last date of storage (D7). Timco was the grape variety that lost the greatest amount of aldehydic compounds (51%), followed by Krissy (35%) and Crimson (6%) (Figure 4). Hexanal and (*E*)-2-hexenal were the most affected (Figure 7). These results do not agree with a previous report on table grapes, in which hexanal and (*E*)-2-hexenal at 5 °C storage and 99% relative humidity increased significantly after 12 weeks of storage [27]. Conversely, the trend of alcohols was different because, after 108 days of storage (15 weeks approximately) (from D4 to D7), the quantity increased in all varieties, with Krissy being the cultivar that exhibited the greatest increase (77%), followed by Timco (58%) and Crimson (53%). These results were mainly due to the increment of hexanol and (*E*)-2-hexenol (Figure 7). When harvested, grapes begin a process of dehydration, and it has been described that alcohol dehydrogenase (ADH) activity increases under this condition [34,35,36]. ADH catalyzes the interconversion of acetaldehyde to ethanol. The C6 aliphatic aldehydes produced during grape ripening stages may be, in turn, reduced by ADH activity to the corresponding alcohols [36,37]. Therefore, this interconversion of hexanal and (*E*)-2-hexenal to hexanol and (*E*)-2-hexenol is produced by the increment of the activity of ADH, partially due to water stress in the berry. Possibly, the higher relative humidity in the study of Matsumoto et al. [27] did not trigger this mechanism to such a high extent, which could partially explain the different results obtained.

Another remarkable tendency was that followed by terpenoids, which decreased in the Crimson (23%) and Krissy (12%) varieties and remained at a nearly constant level in Timco. Aubert et al. described a dramatic loss of linalool in table grape storage at 1 °C for 4 weeks [33]; however, significant differences were not observed between D4 and D7 in the content of linalool in the three monitored varieties (Figure 5). Additionally, focusing on Krissy, which was the variety richer in terpenoids, an increase in the terpene content was observed after 54 DOS (D5) (Figure 4). This increment might have resulted from a higher activity of β-glycosidases, which consequently broke down the β-glycosidic bonds between the sugar and aglycone of the nonaromatic precursor and, therefore, released terpenes into the flesh of the grape in the first weeks postharvest. These compounds later started to decrease at D6 (75 DOS) and decreased below the content existing when harvested at D7 after 108 DOS. 

Octanal and β-ionone, which, according to the findings of Wu et al., are major important drivers of the preference by consumers for table grapes [5], presented similar tendencies. The amounts of octanal and β-ionone gradually decreased from harvest to the end of the storage in the three cultivars. In the case of octanal, Crimson lost 56.1% of the amount present in the berry after harvest, while Timco lost 67.5% and Krissy lost 73.2%. The losses of β-ionone were lesser, with the amount in Crimson grapes diminishing by 8.7%, that in Krissy by 37.7%, and that in Timco by 38.8%. These data showed that the storage period is crucial to the acceptance of the grapes by the consumer (Figure 6).

### 2.5. Multivariate Analysis

**Figure 8:**
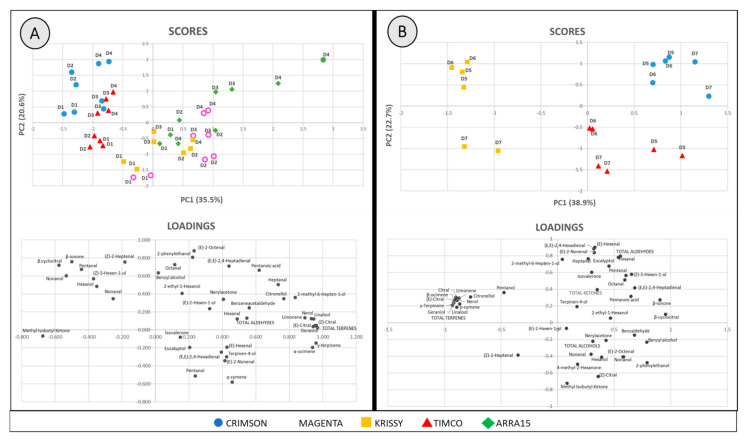
Data scores and loading biplots on the plane of the first two principal components (PC1 against PC2) of (**A**) table grapes along ripening: **D1**, veraison; **D2**, 12 DAV (days after veraison); **D3**, 26 DAV; **D4**, 37 DAV and (**B**) table grapes during storage: **D5**, 54 DOS (days of storage); **D6**, 75 DOS; **D7**, 108 DOS.

For a better understanding of the results obtained, principal component analysis (PCA) was performed using all the volatile compounds and the total sum of each chemical group shown in Table 1 (45 variables). One PCA was performed to study the ripening period employing samples D1–D4 of the five cultivars (Figure 8A) and another one for the storage time with the samples D5–D7 of the three berry varieties studied (Figure 8B). The multivariate analysis comprising the ripening period berries shows the distribution of the grape samples analyzed in the plane defined by PC1 and PC2 (scores) and the variables employed (loadings). The score plot of PC1 and PC2 accounted for 56.2% of the cumulative variance and permitted significant separation of the samples (Figure 8A). The reduction in dimensions showed that Component 1 seemed be related to the grape variety. 

On the other hand, Component 2 seemed to distribute the samples in the plane according to their level of ripening. As previously anticipated, terpenes played an essential role in discriminating among samples. Specifically, on the basis of the loading values, total terpenes (0.968), (*E*)-citral (0.961), γ-terpinene (0.959), geraniol (0.952), linalool (0.945), (*Z*)-citral (0.942), α-ocimene (0.939), nerol (0.932), and limonene (0.893) had a great responsibility in the differentiation of the grape cultivars (Component 1). This can be observed in the loading plot, in which the majority of the terpenes are located in the right half of the plot, where Magenta, Krissy, and Arra15 are also located, which are the varieties that presented higher amounts of these aromatic compounds. However, in the case of discrimination between ripening stages, the statistical results were less defined. It seems that, as reflected in Table S1 (Supplementary Materials), 2-phenylethanol is positively related to ripened berries according to its loading value in the PC2 (0.807) maybe contributing to the increase in floral aroma of this fruit from D1 (veraison) to D4 (37 days after veraison).

The score plots of PC1 and PC2 of the PCA with respect to storage times (Figure 8B) accounted for 61.6% of the cumulative variance and showed that the separation among varieties was more effective and related to PC1. In addition, PC2 seemed to be related to the days of storage and confirmed the stability in storage of Crimson variety, as well as the important instability of Timco, as reflected in Figure 3. Furthermore, this PCA revealed findings not previously perceived because, instead of the conservation of the total amount of volatile compounds of Krissy, the particular quantities of each fluctuated significantly during storage, resulting in the samples after 54 (D5) and 75 (D6) days of storage presenting a very different volatile profile to the samples after 108 days of storage.

## 3. Materials and Methods

### 3.1. Plant Material

*Vitis vinifera* red varieties, Crimson, Magenta™, Krissy™, and Timco™, and the white variety, Arra-15™, grape berries and bunches were collected from a single farm in a commercial vineyard (Huelquen, Santiago, Chile; Latitude/longitude: 33°49′43″ south (S) 70°38′33″ west (W)) during the 2018 season between January to March. All the varieties were seedless. Five vines of each variety were used to carry out a representative sampling, considering five replicates per date and variety. In total, 50 berries per replicate were collected from veraison to commercial maturity every 10 to 12 days. Grapes were collected at four moments (D1, veraison; D2, 12 DAV (days after veraison); D3, 26 DAV (days after veraison); D4, 37 DAV (days after veraison; commercial maturity)) and kept at −20 °C until chemical analysis. After harvesting at D4, the grape bunches were packaged conventionally according to commercial practices and were stored in cold conditions (chamber at 0 °C). During the storage time, samples were taken from the Crimson, Krissy™, and Timco™ varieties on three dates: D5, 54 DOS (days of storage); D6, 75 DOS; D7, 108 DOS. Magenta™ and Arra15™ were not monitored during storage because the other three are the varieties with the higher volume of exportation and, therefore, the most interesting for the company that collaborated in this work with the industrial storage cold chambers. Three replicates (boxes) were considered for each variety on each sampling date. The soluble solids, pH, and acidity were analyzed in 50 berries from different bunches of each box, and 30 berries with pedicel were stored at −20 °C until chromatographic analysis.

### 3.2. Reagents and Standards

The standard compounds employed in this study for identification and quantification were supplied by Sigma-Aldrich (Darmstadt, Germany), including hexanal, (*E*)-2-hexenal, nonanal, (*E*,*E*)-2,4-hexadienal, (*E*)-2-nonenal, benzaldehyde, pentanol, hexanol, (*Z*)-3-hexen-1-ol, (*E*)-2-hexen-1-ol, 2-ethyl-1-hexanol, nonanol, benzyl alcohol, 2-phenylethanol, limonene, α-ocimene, linalool, citronellol, nerol, geraniol, and isovalerone. Sodium chloride and 4-methyl-2-pentanol (internal standard) were purchased from Merck (Darmstadt, Germany).

### 3.3. Berry General Analyses

The soluble solid concentration, pH, and titratable acidity were measured by employing the analytical methods recommended by the International Organization of Vine and Wine [38]. The pH was measured as a function of hydrogen ion concentration by potentiometry (Model Seven Compact S220, Mettler-Toledo Intl. Inc., Columbus, OH, USA), and titratable acidity was measured with 0.1 N NaOH [39]. Lastly, the weight of 50 berries was registered in grams.

### 3.4. Solid-Phase Microextraction (SPME) and Gas Chromatography/Mass Spectrometry Analysis

Grapes were defrosted at ambient temperature over 40 min, and the seeds were removed if present. Afterward, three grapes were crushed in a 50 mL Falcon tube using an Ultra-TurraxTM homogenizer for 1 min. Then, 3.75 g of the sample was placed in a 20 mL glass vial, and 3.75 of milliQ water and 10 μL of 4-methyl-2-pentanol (0.75 mg·L^−1^) (used as an internal standard) were added. For the extraction and analysis, the methodology employed in Ubeda et al. [40] was followed. The extraction was carried out in an MPS Autosampler (Gerstel, Palo Alto, CA, USA), incubating the vial for 20 min at 45 °C with agitation at 500 rpm. Then, the selected SPME fiber was a 2 cm 50/30 μm Carboxen/Divinylbenzene/Polydimethylsiloxane (Supelco, Bellefonte, PA, USA) placed into the headspace of the vial for 40 min. Afterward, the desorption was carried out in the injector in splitless mode for 3 min, with a transfer line temperature of 280 °C. To perform the gas chromatography analysis, a 7890B Agilent GC system coupled to an Agilent 5977 quadrupole inert mass spectrometer (Agilent Technologies, Palo Alto, CA, USA) with a DB Wax capillary column (60 m × 0.25 mm, and 0.25 μm film thickness) (J&W Scientific, Folsom, CA, USA) was used. Helium was used as the carrier gas at a flow rate of 1 mL·min^−1^. The program of the oven was the following: 35 °C for 1 min, followed by an increase to 130 °C at 4.5 °C·min^−1^ with holding for 3 min, an increase to 180 °C at 2.5 °C·min^−1^, and then an increase to 230 °C at 5 °C·min^−1^ with holding for 1 min. The electron ionization mass spectra were recorded in scan mode at 70 eV in the range of 35–300 amu. The MS Chemstation software (Agilent Technologies, Palo Alto, CA, USA) was employed for the recording and processing of the data. Finally, the identification of the compounds was done by employing authentic standards when available, as well as by comparing the mass spectra obtained from each molecule with the reference spectra of the NIST 98 software library (only considering compounds identified with a Match Score higher than 900%) and with the data from the literature. In cases when only the software identified the compound, they were treated as tentatively identified. The data were expressed as the relative area with respect to 4-methyl-2-pentanol (internal standard). The relative concentration was calculated by dividing the peak area of the target ion of each compound by the peak area of the target ion of the internal standard.

### 3.5. Statistical Analysis

Data analysis was carried out using InfoStat (version 2017p, FCA-Universidad Nacional de Córdoba, Argentina). The means were compared using an ANOVA and a post hoc (Tukey) test (α = 0.05). All tests fulfilled the assumptions of residual normality. Principal component analysis (PCA) was performed with IBM SPSS Statistics 26 software (IBM, Barcelona, Spain).

## 4. Conclusions

The changes observed during ripening were much more dramatic than those during storage, with the latter being significant after 108 days of storage only in the case of the Timco variety, in which volatiles decreased, and in the case of Krissy, in which the volatile profile changed. The evolution from veraison to harvest was strongly related to the cultivar in most cases. Only terpenoids reflected a common increasing trend during ripening. Additionally, the terpene composition was highly responsible for the differentiation and characterization of the volatile compounds of the grape varieties studied. Thus, the terpene amounts in Magenta, Krissy, and Arra15 clearly outweighed those in Crimson and Timco. This, together with the fact that Magenta and Arra15 were the varieties with the highest volatile fractions, may point to these varieties as the most aromatically intense among those studied. These results may position the Magenta variety as a very good alternative and competitor to Crimson, which is one of the table grape cultivars in Chile with a high commercial demand currently. These results show strong differences in the volatile evolution and concentration during ripening, as well as during postharvest, which is useful for a better understanding of consumer preferences. Additionally, our results might be of interest for plant breeding programs focusing on the organoleptic properties of new varieties and could better aid in the decision-making concerning planting plans according to the need for storing fresh table grapes given by consumer market distances.

## Figures and Tables

**Table 1:** Volatile compounds determined in the grape cultivars analyzed at their commercial maturity (D4). Data are expressed in relative area (abundance) with respect to the internal standard.

Volatile Compounds	LRI	ID	Aromatic Description	Grape Cultivars				
				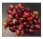 CRIMSON	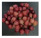 MAGENTA	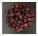 KRISSY	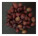 TIMCO	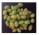 ARRA15
ALDEHYDES								
Pentanal	996	B	Almond/malt/pungent	21.7 ± 3.3 ^b^	13.2 ± 1.4 ^ab^	9.77 ± 2.39 ^a^	11.6 ± 0.6 ^a^	10.4 ± 3.1 ^a^
Hexanal	1093	A	Grass/tallow/fat	474 ± 138 ^b^	572 ± 16 ^b^	212 ± 22 ^a^	414 ± 23 ^ab^	517 ± 27 ^b^
Heptanal	1179	B	Fat/citrus/rancid	5.92 ± 1.75 ^a^	7.15 ± 0.63 ^a^	6.56 ± 1.19 ^a^	6.09 ± 0.06 ^a^	17.5 ± 2.2 ^b^
(*E*)-2-Hexenal	1216	A	Green/leaf	174 ± 1 ^b^	169 ± 21 ^b^	131 ± 11 ^ab^	101 ± 26 ^a^	102 ± 10 ^a^
Octanal	1294	B	Fat/soap/lemon/green	2.16 ± 0.33 ^b^	1.51 ± 0.06 ^a^	1.47 ± 0.08 ^a^	1.48 ± 0.06 ^a^	2.68 ± 0.52 ^b^
(*Z*)-2-Heptenal	1332	B	Pungent/vegetable/soap/fat	14.0 ± 3.2 ^b^	9.71 ± 0.07 ^ab^	5.70 ± 0.16 ^a^	9.87 ± 1.72 ^ab^	8.38 ± 1.42 ^ab^
Nonanal	1397	A	Fat/citrus/green	22.0 ± 1.2 ^b^	15.7 ± 2.9a ^b^	9.82 ± 2.33 ^a^	11.7 ± 4.2 ^ab^	7.86 ± 2.19 ^a^
(*E*,*E*)-2,4-Hexadienal	1418	A	Green	8.14 ± 0.20 ^b^	8.16 ± 0.96 ^b^	6.33 ± 0.69 ^ab^	4.64 ± 0.94 ^a^	4.21 ± 0.77 ^a^
(E)-2-Octenal	1437	A	Green leaf/walnut	25.0 ± 4.3 ^b^	18.8 ± 1.0 ^ab^	10.9 ± 3.3 ^a^	21.6 ± 3.7 ^ab^	30.9 ± 3.3 ^b^
(*E*,*E*)-2-4-Heptadienal	1486	B	Nut/fat	2.35 ± 0.72 ^a^	2.53 ± 0.35 ^a^	1.67 ± 0.09 ^a^	1.94 ± 0.36 ^a^	2.74 ± 0.48 ^a^
(*E*)-2-Nonenal	1548	A	Fatty/green	0.753 ± 0.001 ^a^	1.56 ± 0.45 ^a^	0.849 ± 0.278 ^a^	1.00 ± 0.12 ^a^	1.24 ± 0.06 ^a^
Benzaldehyde	1557	B	Almond/burnt sugar	4.07 ± 0.36 ^a^	4.53 ± 0.81 ^a^	3.37 ± 0.65 ^a^	4.86 ± 0.56 ^a^	4.04 ± 0.47 ^a^
Benzeneacetaldehyde	1660	B	Green/floral/hyacinth	4.16 ± 0.20 ^a^	10.2 ± 3.2 ^a^	4.70 ± 0.29 ^a^	10.1 ± 1.2 ^a^	23.4 ± 2.5 ^b^
ALCOHOLS								
Pentanol	1226	A	Fruit	3.60 ± 0.25 ^a^	90.7 ± 24.6 ^b^	75.9 ± 8.2 ^b^	1.56 ± 0.12 ^a^	3.71 ± 0.76 ^a^
Hexanol	1340	A	Resin/flower/green	140 ± 27 ^a^	103 ± 6 ^a^	72.0 ± 12.9 ^a^	81.3 ± 26.6 ^a^	69.3 ± 7.2 ^a^
(*Z*)-3-Hexen-1-ol	1382	A	Grass	23.9 ± 4.6 ^b^	3.51 ± 0.05 ^a^	4.97 ± 0.99 ^a^	5.82 ± 0.73 ^a^	8.87 ± 0.13 ^a^
(*E*)-2-Hexen-1-ol	1401	A	Green/leaf/walnut	4.44 ± 0.70 ^a^	3.28 ± 0.23 ^a^	3.51 ± 0.48 ^a^	3.54 ± 0.15 ^a^	16.1 ± 2.0 ^b^
2-Methyl-6-heptenol	1429	B	Coriander/green	0.339 ± 0.094 ^a^	0.486 ± 0.087 ^a^	0.459 ± 0.050 ^a^	0.149 ± 0.009 ^a^	2.09 ± 0.44 ^b^
2-Ethyl-1-hexanol	1489	B	Rose/green	11.8 ± 0.9 ^a^	11.2 ± 1.9 ^a^	9.74 ± 2.46 ^a^	11.1 ± 0.2 ^a^	12.9 ± 2.6 ^a^
Nonanol	1659	A	Fat/green	0.910 ± 0.105 ^b^	0.538 ± 0.031 ^a^	0.574 ± 0.160 ^a^	0.429 ± 0.009 ^a^	0.441 ± 0.017 ^a^
Benzyl alcohol	1902	A	Sweet/flower	10.9 ± 2.1 ^b^	3.62 ± 0.08 ^a^	1.52 ± 0.18 ^a^	4.60 ± 0.17 ^a^	3.60 ± 0.35 ^a^
2-Phenylethanol	1931	A	Honey/spice/rose/lilac	4.47 ± 0.97 ^abc^	3.11 ± 0.46 ^ab^	1.60 ± 0.12 ^a^	6.37 ± 1.30 ^bc^	7.77 ± 1.30 ^c^
TERPENES								
Limonene	1191	A	Citrus/mint	3.85 ± 0.01 ^ab^	5.26 ± 0.85 ^b^	4.97 ± 1.26 ^b^	1.47 ± 0.10 ^a^	8.46 ± 0.63 ^c^
Eucalyptol	1198	B	Mint/sweet	1.12 ± 0.07 ^b^	0.870 ± 0.090 ^ab^	1.31 ± 0.21 ^b^	0.609 ± 0.119 ^a^	0.868 ± 0.010 ^ab^
α-Ocimene	1231	A	Fruit/wet clothes	0.447 ± 0.085 ^a^	5.42 ± 0.35 ^b^	6.67 ± 1.29 ^b^	0.558 ± 0.001 ^a^	10.3 ± 1.1 ^c^
*p*-Cymene	1262	B	Fresh citrus/woody spice	1.34 ± 0.18 ^a^	4.42 ± 0.09 ^b^	5.06 ± 0.92 ^b^	0.998 ± 0.088 ^a^	4.86 ± 0.64 ^b^
γ-Terpinene	1287	B	Gasoline/turpentine	nd ^a^	0.836 ± 0.048 ^b^	0.843 ± 0.220 ^b^	0.052 ± 0.011 ^a^	1.28 ± 0.17 ^b^
Linalool	1541	A	Flower/lavender	0.328 ± 0.099 ^a^	5.96 ± 0.84 ^bc^	3.20 ± 0.90 ^ab^	0.436 ± 0.039 ^a^	9.98 ± 2.71 ^c^
Terpinen-4-ol	1626	B	Turpentine/nutmeg/must	0.313 ± 0.031 ^b^	0.429 ± 0.058 ^b^	0.309 ± 0.016 ^b^	nd ^a^	0.563 ± 0.022 ^c^
β-Cyclocitral	1650	B	Mint	1.12 ± 0.14 ^c^	0.594 ± 0.020 ^ab^	0.356 ± 0.029 ^a^	0.935 ± 0.167 ^bc^	0.553 ± 0.012 ^ab^
(*Z*)-Citral	1671	B	Lemon	nd ^a^	1.12 ± 0.19 ^bc^	1.14 ± 0.04 ^c^	0.150 ± 0.009 ^ab^	2.05 ± 0.51 ^c^
(*E*)-Citral	1699	B	Citrus	0.338 ± 0.050 ^a^	6.11 ± 0.51 ^b^	8.09 ± 0.17 ^bc^	0.585 ± 0.061 ^a^	11.6 ± 2.45 ^c^
Citronellol	1722	A	Rose	0.337 ± 0.044 ^a^	0.545 ± 0.087 ^a^	0.697 ± 0.070 ^a^	0.188 ± 0.013 ^a^	3.64 ± 1.46 ^b^
Nerol	1804	A	Sweet	1.25 ± 0.03 ^a^	18.9 ± 1.7 ^ab^	16.2 ± 1.1 ^ab^	0.511 ± 0.044 ^a^	31.9 ± 15.1 ^b^
Geraniol	1852	A	Rose/geranium	2.49 ± 0.47 ^a^	66.1 ± 8.1 ^bc^	78.1 ± 21.1 ^c^	4.17 ± 0.03 ^ab^	127 ± 26 ^c^
Geranyl acetone	1859	C	Magnolia/green	0.992 ± 0.125 ^a^	1.37 ± 0.15 ^a^	0.802 ± 0.055 ^a^	0.890 ± 0.224 ^a^	0.930 ± 0.200 ^a^
KETONES								
Methyl isobutyl ketone	1010	C	Green/herbal/fruity	10.8 ± 0.1 ^a^	10.1 ± 0.4 ^a^	10.6 ± 0.1 ^a^	11.2 ± 0.2 ^a^	9.9 ± 0.7 ^a^
4-Methyl-2-hexanone	1129	B	-	4.39 ± 0.28 ^a^	4.26 ± 0.27 ^a^	4.49 ± 0.16 ^a^	4.51 ± 0.03 ^a^	4.83 ± 0.19 ^a^
Isovalerone	1195	A	Mild, sweet odor	40.1 ± 1.9 ^a^	36.1 ± 6.8 ^a^	40.5 ± 0.9 ^a^	34.4 ± 5.8 ^a^	41.1 ± 4.1 ^a^
C13-NORISOPRENOIDS								
β-Ionone	1951	A	Violet, flower, raspberry	3.29 ± 0.80 ^c^	1.53 ± 0.12 ^ab^	1.05 ± 0.04 ^a^	1.48 ± 0.11 ^ab^	2.40 ± 0.51 ^bc^
ACIDS								
Pentanoic acid	1861	C	Cheesy	8.92 ± 0.08 ^a^	8.56 ± 0.06 ^a^	7.7 ± 1.2 ^a^	10.1 ± 2.4 ^a^	19.1 ± 6.1 ^a^

Values with different superscript letters indicate statistically significant differences (*p* < 0.05). LRI, linear retention index. ID, reliability of identification: A, mass spectrum and LRI agreed with standards; B, mass spectrum agreed with mass spectral data base and LRI agreed with the literature data; C, tentatively identified, mass spectrum agreed with mass spectral database. Aromatic description from Flavornet (https://www.flavornet.org/).

## Supplementary Materials

The following are available online, Figure S1: Chromatogram of one of the grape berries and Table S1: Volatile compounds evolution along maturity and storage. Different letter indicates statistically significant differences among the sampling times D1-D7 (*p* < 0.05). Sampling along harvest D1: veraison; D2: 12 DAV (days after veraison), D3: 26 DAV; D4: 37 DAV. Sampling along storage: D5: 54 DOS (days of storage), D6: 75 DOS and D7: 108 DOS.
