# Supplementary material for: Ripening and Storage Time Effects on the Aromatic Profile of New Table Grape Cultivars in Chile
Source: Molecules. 2020 Dec 8;25(24):5790. doi: 10.3390/molecules25245790 (PMC7763542; doi:10.3390/molecules25245790)
Supplement: Supplementary file 1 [file molecules-25-05790-s001.pdf]

Supplementary material

Ripening and storage time effects on the aromatic profile of new table grape cultivars in Chile

Cristina Ubeda [1,2], Mariona Gil i Cortiella [3], Luis Villalobos-González [4], Camila Gómez [5], Claudio Pastenes [4], and Álvaro Peña Neira [5,*]

[1] Departamento de Nutrición y Bromatología, Toxicología y Medicina Legal, Facultad de Farmacia, Universidad de Sevilla, C/Profesor García González 2, 41012 Sevilla, Spain; c_ubeda@us.es
[2] Instituto de Ciencias Biomédicas, Facultad de Ciencias, Universidad Autónoma de Chile, Chile; cristina.ubeda@uautonoma.cl
[3] Instituto de Ciencias Químicas Aplicadas, Facultad de Ingeniería, Universidad Autónoma de Chile, Chile; Mariona.gil@uautonoma.cl
[4] Departamento de Producción Agrícola, Facultad de Ciencias Agronómicas, Universidad de Chile, Santiago, Chile; cpastene@uchile.cl
[5] Department of Agro-Industry and Enology, Faculty of Agronomical Sciences, University of Chile, Post Office Box 1004, Santa Rosa 11315, La Pintana, Santiago, Chile; apena@uchile.cl
* Correspondence: apena@uchile.cl

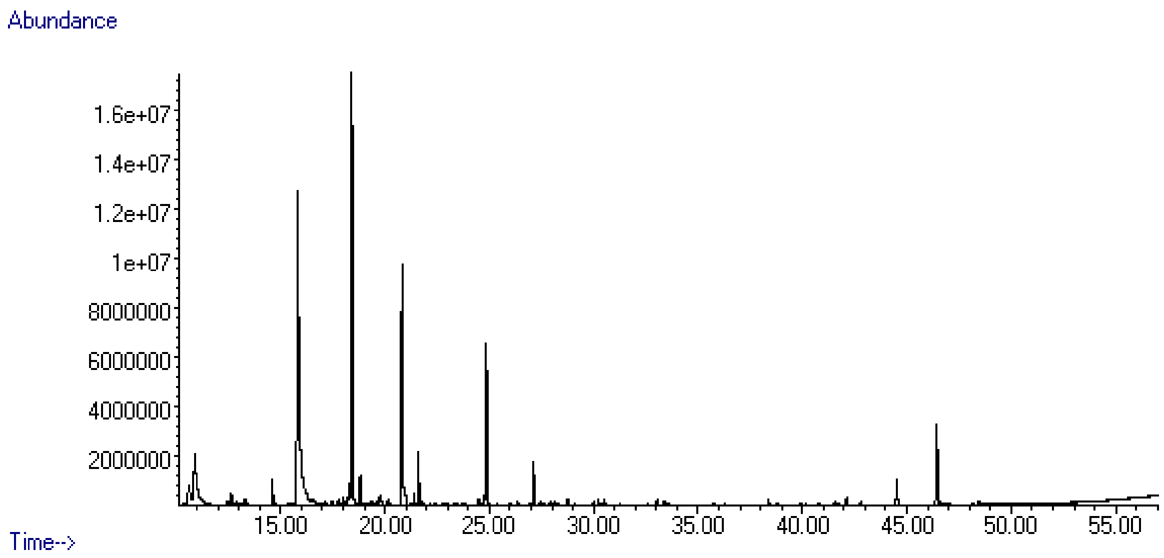

Figure S1. Chromatogram of one of the grape berries.

Table S1. Volatile compounds evolution along maturity and storage. Different letter indicates statistically significant differences among the sampling times D1-D7 ($p < 0.05$). Sampling along harvest **D1**: veraison; **D2**: 12 DAV (days after veraison), **D3**: 26 DAV; **D4**: 37 DAV. Sampling along storage: **D5**: 54 DOS (days of storage), **D6**: 75 DOS and **D7**: 108 DOS.

		**RIPENING GRAPE SAMPLES**				**STORAGE GRAPE SAMPLES**		
		**D1**	**D2**	**D3**	**D4**	**D5**	**D6**	**D7**
**Compound**	**variety**	mean ± SD	mean ± SD	mean ± SD	mean ± SD	mean ± SD	mean ± SD	mean ± SD
Pentanal	CRIMSON	19.8 ± 1.9	21.7 ± 5.2	14.8 ± 0.2	21.7 ± 3.3	12.2 ± 2.3	14.7 ± 0.8	13.5 ± 1.2
	KRISSY	11.9 ± 4.1ab	9.81 ± 1.15ab	12.7 ± 2.5b	9.77 ± 2.39ab	10.9 ± 1.5ab	5.44 ± 1.24ab	3.67 ± 0.39a
	TIMCO	8.81 ± 1.21ab	8.74 ± 0.09ab	12.7 ± 0.2c	11.6 ± 0.6bc	8.26 ± 1.08ab	10.3 ± 1.7bc	6.03 ± 0.51a
	MAGENTA	9.51 ± 0.31ab	8.52 ± 1.01a	9.27 ± 1.28ab	13.2 ± 1.4b	– –	– –	– –
	ARRA15	13.2 ± 0.3	10.3 ± 1.2	12.2 ± 1.2	10.4 ± 3.1	– –	– –	– –
Heptanal	CRIMSON	3.23 ± 0.76	2.64 ± 0.15	4.26 ± 0.16	5.92 ± 1.75	6.13 ± 1.01	5.33 ± 0.84	4.84 ± 1.59
	KRISSY	2.58 ± 0.37a	3.03 ± 0.33a	3.75 ± 0.16a	6.56 ± 1.19b	3.49 ± 0.61a	4.00 ± 0.15a	2.67 ± 0.26a
	TIMCO	2.32 ± 0.26ab	3.00 ± 0.76b	3.06 ± 0.24b	6.09 ± 0.06c	2.68 ± 0.54b	5.20 ± 0.63c	0.56 ± 0.16a
	MAGENTA	2.34 ± 0.03a	3.88 ± 0.23ab	5.35 ± 0.78bc	7.15 ± 0.63c	– –	– –	– –
	ARRA15	4.65 ± 0.49a	9.48 ± 1.17ab	14.40 ± 2.54bc	17.47 ± 2.23c	– –	– –	– –
(Z)-2-Heptenal	CRIMSON	11.0 ± 2.0ab	19.0 ± 2.9c	9.77 ± 0.12ab	14.0 ± 3.2bc	3.89 ± 0.20a	5.84 ± 1.32a	9.31 ± 1.75ab
	KRISSY	2.91 ± 0.18a	7.98 ± 1.17c	5.95 ± 0.63bc	5.70 ± 0.16bc	4.74 ± 0.23ab	3.91 ± 1.34ab	69.22 ± 0.44d
	TIMCO	4.80 ± 0.79ab	4.04 ± 1.02ab	8.44 ± 0.39bc	9.87 ± 1.72c	10.5 ± 2.2c	4.67 ± 1.15ab	3.41 ± 0.51a
	MAGENTA	4.57 ± 0.04a	4.09 ± 0.88a	6.95 ± 0.54b	9.71 ± 0.07c	– –	– –	– –
	ARRA15	10.0 ± 1.4	11.4 ± 3.9	10.6 ± 2.3	8.38 ± 1.42	– –	– –	– –
Nonanal	CRIMSON	5.81 ± 0.22a	14.3 ± 0.3bc	22.4 ± 2.7c	22.0 ± 1.2bc	14.3 ± 1.3bc	13.7 ± 1.3ab	20.7 ± 4.4bc
	KRISSY	9.61 ± 2.70a	10.3 ± 2.0a	14.0 ± 1.8a	9.82 ± 2.33a	9.86 ± 0.33a	12.1 ± 2.3a	24.2 ± 2.1b
	TIMCO	13.8 ± 1.8a	12.8 ± 3.3a	28.3 ± 4.1b	11.7 ± 4.2a	31.3 ± 5.8b	5.91 ± 0.39a	13.1 ± 1.1a
	MAGENTA	4.27 ± 0.95a	9.97 ± 3.14ab	25.8 ± 0.2c	15.7 ± 2.9b	– –	– –	– –
	ARRA15	9.27 ± 2.22	7.17 ± 0.65	10.5 ± 2.0	7.86 ± 2.19	– –	– –	– –
(E.E)-2,4-Hexadienal	CRIMSON	1.96 ± 0.06a	2.87 ± 0.97ab	7.71 ± 1.59c	8.14 ± 0.20c	8.14 ± 0.48c	5.81 ± 1.61bc	7.34 ± 0.48c

	KRISSY	4.14 ± 0.54b	8.35 ± 0.58d	5.96 ± 1.12bc	6.33 ± 0.69cd	3.92 ± 0.34ab	6.89 ± 0.14cd	1.83 ± 0.31a
	TIMCO	5.79 ± 0.22d	3.85 ± 0.05abc	5.36 ± 0.01cd	4.64 ± 0.94bcd	3.08 ± 0.79ab	3.38 ± 0.16ab	2.00 ± 0.05a
	MAGENTA	7.28 ± 0.28a	10.5 ± 0.0a	14.5 ± 1.5b	8.16 ± 0.96a	– –	– –	– –
	ARRA15	6.53 ± 0.52ab	8.40 ± 1.46b	5.55 ± 0.36ab	4.21 ± 0.77a	– –	– –	– –
(E.E)-2,4-Heptadienal	CRIMSON	1.78 ± 0.39	2.21 ± 0.15	1.89 ± 0.02	2.35 ± 0.72	1.90 ± 0.13	1.91 ± 0.30	2.82 ± 0.30
	KRISSY	1.21 ± 0.33	1.74 ± 0.12	1.57 ± 0.19	1.67 ± 0.09	1.49 ± 0.20	1.50 ± 0.03	0.568 ± 0.054
	TIMCO	1.55 ± 0.02ab	1.52 ± 0.24ab	1.89 ± 0.12ab	1.94 ± 0.36b	2.15 ± 0.30b	1.98 ± 0.29b	0.952 ± 0.223a
	MAGENTA	1.20 ± 0.18a	1.73 ± 0.13ab	2.34 ± 0.30b	2.53 ± 0.35b	– –	– –	– –
	ARRA15	1.93 ± 0.01	2.36 ± 0.06	2.63 ± 0.30	2.74 ± 0.48	– –	– –	– –
(E)-2-Octenal	CRIMSON	16.5 ± 3.1ab	30.3 ± 3.0c	16.9 ± 3.8ab	25.0 ± 4.3bc	11.7 ± 0.6a	18.1 ± 1.3ab	24.7 ± 1.9bc
	KRISSY	6.4 ± 1.1ab	15.3 ± 1.1b	11.9 ± 2.2b	10.9 ± 3.3b	10.3 ± 0.9b	10.5 ± 2.0b	13.6 ± 1.6a
	TIMCO	9.7 ± 0.2a	9.6 ± 1.6a	19.6 ± 3.9ab	21.6 ± 3.7b	42.3 ± 1.7c	14.6 ± 4.1ab	17.2 ± 1.9ab
	MAGENTA	7.99 ± 1.02a	10.2 ± 0.8a	14.3 ± 0.3b	18.8 ± 1.0c	– –	– –	– –
	ARRA15	17.5 ± 1.2a	24.4 ± 2.6ab	28.7 ± 0.4b	30.9 ± 3.3b	– –	– –	– –
(E)-2-Nonenal	CRIMSON	0.743 ± 0.212	0.871 ± 0.122b	0.936 ± 0.170ab	0.753 ± 0.001ab	1.35 ± 0.19ab	1.59 ± 0.13ab	1.71 ± 0.64b
	KRISSY	0.582 ± 0.108a	1.75 ± 0.16c	0.886 ± 0.051ab	0.849 ± 0.278a	0.888 ± 0.094ab	1.40 ± 0.21bc	0.459 ± 0.048
	TIMCO	2.52 ± 0.11	0.826 ± 0.100	1.02 ± 0.41	1.00 ± 0.12	0.548 ± 0.076	0.846 ± 0.102	0.623 ± 0.140
	MAGENTA	3.02 ± 0.28ab	4.84 ± 1.11b	2.90 ± 0.25ab	1.56 ± 0.45a	– –	– –	– –
	ARRA15	0.962 ± 0.204a	2.02 ± 0.50ab	3.11 ± 0.57c	1.24 ± 0.06a	– –	– –	– –
Benzaldehyde	CRIMSON	1.78 ± 0.11a	2.48 ± 0.53ab	3.52 ± 1.18ab	4.07 ± 0.36b	3.51 ± 0.09ab	2.48 ± 0.07ab	3.38 ± 0.33ab
	KRISSY	6.05 ± 1.20b	3.69 ± 0.38ab	3.86 ± 0.51ab	3.37 ± 0.65a	2.73 ± 0.21a	2.02 ± 0.34a	1.68 ± 0.08a
	TIMCO	8.88 ± 0.02c	3.17 ± 0.18ab	5.77 ± 1.61b	4.86 ± 0.56ab	4.42 ± 0.05ab	3.64 ± 0.15ab	2.77 ± 0.50a
	MAGENTA	3.03 ± 0.17	2.81 ± 0.24	4.84 ± 1.04	4.53 ± 0.81	– –	– –	– –
	ARRA15	3.21 ± 0.49ab	2.09 ± 0.00a	4.26 ± 0.02b	4.04 ± 0.47b	– –	– –	– –
Benzene acetaldehyde	CRIMSON	2.09 ± 0.13a	4.08 ± 0.26abc	3.90 ± 1.34ab	4.16 ± 0.20abc	5.87 ± 0.38bc	11.9 ± 0.7d	6.41 ± 0.47c
	KRISSY	3.54 ± 0.71a	4.20 ± 0.33ab	4.38 ± 1.26ab	4.70 ± 0.29ab	5.83 ± 0.11b	5.30 ± 0.50ab	6.11 ± 0.18b
	TIMCO	12.4 ± 0.8	8.60 ± 0.07	16.4 ± 5.0	10.1 ± 1.2	16.3 ± 4.5	10.4 ± 0.1	21.3 ± 7.0

	MAGENTA	7.84 ± 2.14	13.8 ± 0.6	9.23 ± 2.47	10.2 ± 3.2	– –	– –	– –
	ARRA15	7.79 ± 2.10a	7.70 ± 1.84a	13.1 ± 0.7a	23.4 ± 2.5b	– –	– –	– –
Pentanol	CRIMSON	2.58 ± 0.08a	2.87 ± 0.06a	104 ± 2b	3.60 ± 0.25a	3.23 ± 0.51a	3.65 ± 0.28a	4.21 ± 0.08a
	KRISSY	1.63 ± 0.27a	101 ± 2c	82.5 ± 0.2b	75.9 ± 8.2b	1.63 ± 0.25a	83.7 ± 11.1b	2.94 ± 0.30a
	TIMCO	77.1 ± 9.9b	62.1 ± 17.5b	70.7 ± 1.0b	1.56 ± 0.12a	2.88 ± 0.54a	1.45 ± 0.16a	2.65 ± 0.76a
	MAGENTA	82.6 ± 13.3a	121 ± 2ab	169 ± 19b	90.7 ± 24.6a	– –	– –	– –
	ARRA15	83.8 ± 1.9b	111 ± 9c	3.08 ± 0.30a	3.71 ± 0.76a	– –	– –	– –
(Z)-3-Hexen-1-ol	CRIMSON	10.4 ± 1.3a	16.7 ± 4.2ab	22.2 ± 2.3ab	23.9 ± 4.6b	16.4 ± 3.9ab	15.1 ± 3.2ab	10.9 ± 2.1a
	KRISSY	6.58 ± 0.61bc	6.17 ± 0.72bc	7.32 ± 0.61c	4.97 ± 0.99ab	3.55 ± 0.21a	3.42 ± 0.37a	3.03 ± 0.00a
	TIMCO	10.4 ± 2.5b	6.40 ± 1.31ab	7.75 ± 1.75ab	5.82 ± 0.73ab	5.03 ± 1.11ab	3.02 ± 0.78a	3.63 ± 0.49a
	MAGENTA	2.54 ± 0.21a	2.87 ± 0.31a	4.59 ± 0.45b	3.51 ± 0.05ab	– –	– –	– –
	ARRA15	15.9 ± 4.7	10.6 ± 0.4	9.34 ± 0.48	8.87 ± 0.13	– –	– –	– –
2-methyl-6-Hepten-1-ol	CRIMSON	0.174 ± 0.022a	0.307 ± 0.040ab	0.356 ± 0.053ab	0.339 ± 0.094ab	0.479 ± 0.070ab	0.508 ± 0.131b	0.613 ± 0.078b
	KRISSY	0.213 ± 0.029	0.499 ± 0.133	0.392 ± 0.066	0.459 ± 0.050	0.527 ± 0.167	0.378 ± 0.017	0.372 ± 0.039
	TIMCO	n.d. a	n.d. a	n.d. a	0.149 ± 0.009b	0.175 ± 0.034b	0.151 ± 0.005b	0.157 ± 0.023b
	MAGENTA	0.208 ± 0.010a	0.538 ± 0.037b	0.474 ± 0.040b	0.486 ± 0.087b	– –	– –	– –
	ARRA15	0.579 ± 0.061a	1.16 ± 0.36ab	1.21 ± 0.05ab	2.09 ± 0.44b	– –	– –	– –
2-ethyl-1-Hexanol	CRIMSON	4.99 ± 1.53a	4.79 ± 0.08a	10.5 ± 1.4b	11.8 ± 0.9b	17.0 ± 0.6c	9.47 ± 0.89b	19.9 ± 0.2c
	KRISSY	4.16 ± 0.72a	5.65 ± 0.66a	14.1 ± 3.0c	9.74 ± 2.46abc	15.7 ± 1.3c	7.50 ± 0.02ab	12.3 ± 2.8bc
	TIMCO	4.89 ± 0.28a	14.4 ± 0.6ab	16.8 ± 3.1b	11.1 ± 0.2ab	19.0 ± 5.8b	8.80 ± 1.90ab	10.3 ± 1.5ab
	MAGENTA	6.67 ± 1.73ab	4.84 ± 0.23a	14.0 ± 0.3c	11.2 ± 1.9bc	– –	– –	– –
	ARRA15	4.33 ± 0.25a	5.47 ± 1.04ab	13.9 ± 2.7c	12.9 ± 2.6bc	– –	– –	– –
Benzyl alcohol	CRIMSON	1.24 ± 0.03a	1.33 ± 0.21a	1.52 ± 0.41a	10.89 ± 2.09c	4.60 ± 0.29ab	3.17 ± 0.24ab	7.05 ± 2.01bc
	KRISSY	1.13 ± 0.14a	1.38 ± 0.40ab	2.25 ± 0.39b	1.52 ± 0.18ab	1.72 ± 0.36ab	1.49 ± 0.13ab	2.23 ± 0.19b
	TIMCO	1.36 ± 0.04a	1.94 ± 0.42a	2.51 ± 0.14ab	4.60 ± 0.17c	4.32 ± 0.34bc	4.54 ± 1.03c	5.73 ± 0.62c
	MAGENTA	1.19 ± 0.34a	1.28 ± 0.09a	2.39 ± 0.40b	3.62 ± 0.08c	– –	– –	– –
	ARRA15	1.83 ± 0.15a	2.21 ± 0.18a	4.14 ± 0.23b	3.60 ± 0.35b	– –	– –	– –

2-phenylethanol	CRIMSON	1.88 ± 0.54a	4.89 ± 0.88abc	3.14 ± 1.04ab	4.47 ± 0.97abc	6.39 ± 1.58bc	5.79 ± 0.39abc	8.68 ± 1.72c
	KRISSY	0.716 ± 0.109a	1.13 ± 0.13ab	1.26 ± 0.29ab	1.60 ± 0.12bc	2.63 ± 0.13d	2.22 ± 0.62cd	2.89 ± 0.02d
	TIMCO	2.29 ± 0.73a	2.16 ± 0.04a	3.42 ± 0.47ab	6.37 ± 1.30bc	11.88 ± 1.71d	6.44 ± 0.35bc	10.00 ± 0.97cd
	MAGENTA	1.20 ± 0.23a	2.46 ± 0.22ab	1.75 ± 0.41ab	3.11 ± 0.46b	− −	− −	− −
	ARRA15	1.62 ± 0.07a	2.95 ± 0.75a	4.39 ± 0.24a	7.77 ± 1.30b	− −	− −	− −
Nonanol	CRIMSON	1.08 ± 0.13bc	1.25 ± 0.05c	0.730 ± 0.192abc	0.910 ± 0.105abc	0.410 ± 0.000a	0.593 ± 0.056ab	0.709 ± 0.266abc
	KRISSY	0.470 ± 0.095	0.425 ± 0.071	0.503 ± 0.188	0.574 ± 0.160	0.394 ± 0.118	0.317 ± 0.108	0.513 ± 0.004
	TIMCO	0.480 ± 0.072abc	0.501 ± 0.001abc	0.736 ± 0.083c	0.429 ± 0.009ab	0.738 ± 0.119c	0.341 ± 0.008a	0.665 ± 0.076bc
	MAGENTA	0.327 ± 0.012a	0.349 ± 0.025ab	0.477 ± 0.052bc	0.538 ± 0.031c	− −	− −	− −
	ARRA15	0.511 ± 0.030	0.471 ± 0.007	0.577 ± 0.097	0.441 ± 0.017	− −	− −	− −
Limonene	CRIMSON	0.730 ± 0.024a	0.980 ± 0.036ab	1.90 ± 0.07abc	3.85 ± 0.01d	2.22 ± 0.09bc	2.87 ± 0.56cd	1.80 ± 0.78abc
	KRISSY	2.68 ± 0.20a	4.28 ± 0.58ab	3.20 ± 0.19a	4.97 ± 1.26ab	6.76 ± 1.07bc	7.91 ± 1.08c	4.87 ± 0.76ab
	TIMCO	1.65 ± 0.38	2.92 ± 0.75	1.61 ± 0.09	1.47 ± 0.10	2.50 ± 0.44	2.03 ± 0.04	2.25 ± 0.22
	MAGENTA	2.23 ± 0.11a	5.36 ± 0.61b	4.41 ± 0.17b	5.26 ± 0.85b	− −	− −	− −
	ARRA15	2.27 ± 0.08a	3.52 ± 0.54ab	4.43 ± 0.63b	8.46 ± 0.63c	− −	− −	− −
Eucalyptol	CRIMSON	1.45 ± 0.30b	0.423 ± 0.163a	1.45 ± 0.11b	1.12 ± 0.07ab	0.939 ± 0.256ab	0.972 ± 0.005ab	0.809 ± 0.242ab
	KRISSY	0.635 ± 0.165b	1.25 ± 0.03cd	0.88 ± 0.15bc	1.31 ± 0.21d	0.74 ± 0.02b	0.17 ± 0.02a	0.08 ± 0.01a
	TIMCO	0.961 ± 0.082c	0.662 ± 0.073bc	0.890 ± 0.135bc	0.609 ± 0.119b	0.244 ± 0.013a	0.164 ± 0.000a	0.206 ± 0.032a
	MAGENTA	0.775 ± 0.070a	1.72 ± 0.35ab	3.07 ± 0.62b	0.870 ± 0.090a	− −	− −	− −
	ARRA15	4.53 ± 0.60b	1.86 ± 0.04a	0.826 ± 0.115a	0.868 ± 0.010a	− −	− −	− −
α-ocimene	CRIMSON	0.300 ± 0.015	0.277 ± 0.017	0.393 ± 0.089	0.447 ± 0.085	0.514 ± 0.056	0.449 ± 0.059	0.465 ± 0.152
	KRISSY	5.20 ± 0.92ab	7.72 ± 1.17ab	4.43 ± 0.12a	6.67 ± 1.29ab	8.13 ± 1.28aba	8.97 ± 0.83b	5.77 ± 1.84ab
	TIMCO	0.221 ± 0.027a	0.471 ± 0.156abc	0.332 ± 0.031ab	0.558 ± 0.008bc	0.668 ± 0.046c	0.526 ± 0.071bc	0.565 ± 0.030bc
	MAGENTA	3.91 ± 0.45a	6.85 ± 0.58b	4.87 ± 0.19a	5.42 ± 0.35ab	− −	− −	− −
	ARRA15	4.60 ± 0.03a	6.29 ± 1.70ab	6.50 ± 1.42ab	10.3 ± 1.1b	− −	− −	− −
p-cymene	CRIMSON	2.29 ± 0.20b	1.41 ± 0.25a	1.11 ± 0.01a	1.34 ± 0.18a	1.09 ± 0.19a	1.14 ± 0.09a	0.944 ± 0.110a
	KRISSY	8.43 ± 1.57b	5.49 ± 1.72ab	2.90 ± 0.07a	5.06 ± 0.92ab	2.99 ± 0.95a	4.36 ± 0.94ab	2.92 ± 0.24a

	TIMCO	1.73 ± 0.13b	1.24 ± 0.25ab	0.938 ± 0.029a	0.998 ± 0.088a	1.21 ± 0.25ab	1.06 ± 0.07a	1.13 ± 0.17ab
	MAGENTA	10.3 ± 1.1b	6.69 ± 0.08a	4.45 ± 0.47a	4.42 ± 0.09a	– –	– –	– –
	ARRA15	10.2 ± 0.9b	5.54 ± 1.27a	4.70 ± 0.74a	4.86 ± 0.64a	– –	– –	– –
γ-Terpinene	CRIMSON	n.d. a	n.d. a	n.d. a	n.d. a	0.101 ± 0.008b	0.081 ± 0.022b	0.126 ± 0.031b
	KRISSY	0.541 ± 0.098a	0.826 ± 0.128ab	0.482 ± 0.001a	0.843 ± 0.220ab	1.35 ± 0.21c	1.05 ± 0.11bc	0.741 ± 0.162ab
	TIMCO	n.d. a	0.111 ± 0.001e	0.0357 ± 0.0072b	0.0516 ± 0.0108bc	0.0854 ± 0.0033d	0.0801 ± 0.0077d	0.0747 ± 0.0005cd
	MAGENTA	0.401 ± 0.115a	0.945 ± 0.080b	0.652 ± 0.062ab	0.836 ± 0.048b	– –	– –	– –
	ARRA15	0.465 ± 0.005a	0.699 ± 0.213a	0.905 ± 0.014ab	1.28 ± 0.17b	– –	– –	– –
Linalool	CRIMSON	n.d. a	n.d. a	n.d. a	0.328 ± 0.099b	0.305 ± 0.061b	0.295 ± 0.017b	0.321 ± 0.107b
	KRISSY	1.75 ± 0.28a	3.61 ± 0.17ab	2.20 ± 0.18a	3.20 ± 0.90ab	4.77 ± 0.94b	3.31 ± 0.85ab	3.06 ± 0.74ab
	TIMCO	0.206 ± 0.044a	0.205 ± 0.005a	0.256 ± 0.017a	0.436 ± 0.039bc	0.520 ± 0.097c	0.275 ± 0.020ab	0.330 ± 0.022ab
	MAGENTA	0.979 ± 0.222a	3.99 ± 0.25b	5.32 ± 0.68b	5.96 ± 0.84b	– –	– –	– –
	ARRA15	1.69 ± 0.41a	3.46 ± 1.25a	4.23 ± 0.18ab	9.98 ± 2.71b	– –	– –	– –
Terpinen-4-ol	CRIMSON	0.338 ± 0.046ab	0.359 ± 0.028b	0.207 ± 0.038ab	0.313 ± 0.031ab	0.172 ± 0.002a	0.206 ± 0.013ab	0.332 ± 0.084ab
	KRISSY	0.856 ± 0.192b	0.635 ± 0.187ab	0.313 ± 0.054a	0.309 ± 0.016a	0.225 ± 0.057a	0.236 ± 0.006a	0.224 ± 0.015a
	TIMCO	0.044 ± 0.063a	n.d. a	n.d. a	n.d. a	0.424 ± 0.087b	n.d. a	n.d. a
	MAGENTA	0.927 ± 0.166b	0.689 ± 0.089ab	0.480 ± 0.048a	0.429 ± 0.058a	– –	– –	– –
	ARRA15	1.64 ± 0.05b	0.875 ± 0.157a	0.716 ± 0.072a	0.563 ± 0.022a	– –	– –	– –
β-cyclocitral	CRIMSON	0.990 ± 0.075abc	1.17 ± 0.02c	0.969 ± 0.130abc	1.12 ± 0.14bc	0.723 ± 0.087a	0.791 ± 0.041ab	1.07 ± 0.05abc
	KRISSY	0.279 ± 0.067	0.400 ± 0.050	0.388 ± 0.013	0.356 ± 0.029	0.255 ± 0.050	0.314 ± 0.015	0.265 ± 0.020
	TIMCO	0.798 ± 0.212	0.704 ± 0.006	0.917 ± 0.090	0.935 ± 0.167	0.829 ± 0.043	0.534 ± 0.012	0.582 ± 0.143
	MAGENTA	0.345 ± 0.009a	0.449 ± 0.041ab	0.609 ± 0.075b	0.594 ± 0.020b	– –	– –	– –
	ARRA15	0.550 ± 0.011	0.523 ± 0.004	0.629 ± 0.106	0.553 ± 0.012	– –	– –	– –
(Z)-Citral	CRIMSON	n.d. a	n.d. a	n.d. a	n.d. a	0.124 ± 0.005b	n.d. a	n.d. a
	KRISSY	0.364 ± 0.051a	1.09 ± 0.18bc	0.759 ± 0.013ab	1.14 ± 0.04bc	1.37 ± 0.23c	1.49 ± 0.15c	0.792 ± 0.099ab
	TIMCO	n.d. a	n.d. a	n.d. a	0.150 ± 0.009b	n.d. a	n.d. a	n.d. a
	MAGENTA	0.286 ± 0.077a	0.899 ± 0.165b	1.00 ± 0.05b	1.12 ± 0.19b	– –	– –	– –

	ARRA15	0.401 ± 0.027a	0.812 ± 0.235a	1.19 ± 0.23ab	2.05 ± 0.51b	− −	− −	− −
(E)-Citral	CRIMSON	n.d. a	n.d. a	0.226 ± 0.028b	0.338 ± 0.050b	0.412 ± 0.029c	0.316 ± 0.014b	0.369 ± 0.042c
	KRISSY	2.52 ± 0.20a	5.91 ± 0.79bc	4.69 ± 0.29ab	8.09 ± 0.17c	7.39 ± 0.02c	7.82 ± 0.44c	4.61 ± 1.67ab
	TIMCO	0.186 ± 0.054a	0.284 ± 0.061ab	0.339 ± 0.005ab	0.585 ± 0.061c	0.432 ± 0.050bc	0.387 ± 0.067abc	0.410 ± 0.077abc
	MAGENTA	1.74 ± 0.31a	4.63 ± 0.77b	5.48 ± 0.19b	6.11 ± 0.51b	− −	− −	− −
	ARRA15	2.16 ± 0.44a	4.65 ± 1.25a	6.88 ± 1.43ab	11.6 ± 2.5b	− −	− −	− −
Citronellol	CRIMSON	0.320 ± 0.006ab	0.496 ± 0.046b	0.220 ± 0.088a	0.337 ± 0.044ab	0.309 ± 0.030ab	0.255 ± 0.022ab	0.434 ± 0.118ab
	KRISSY	0.326 ± 0.023a	0.778 ± 0.116b	0.277 ± 0.029a	0.697 ± 0.070b	1.78 ± 0.13c	0.964 ± 0.004b	0.802 ± 0.192b
	TIMCO	0.106 ± 0.007a	0.177 ± 0.035ab	0.176 ± 0.012ab	0.188 ± 0.013ab	0.205 ± 0.036b	0.146 ± 0.019ab	0.230 ± 0.027b
	MAGENTA	0.301 ± 0.080	0.661 ± 0.174	0.459 ± 0.081	0.545 ± 0.087	− −	− −	− −
	ARRA15	1.44 ± 0.13	2.19 ± 0.35	2.67 ± 0.17	3.64 ± 1.46	− −	− −	− −
Neryl acetone	CRIMSON	0.437 ± 0.097a	1.07 ± 0.23ab	0.877 ± 0.096ab	0.992 ± 0.125ab	0.685 ± 0.003ab	1.02 ± 0.01ab	1.30 ± 0.35b
	KRISSY	0.799 ± 0.168	1.19 ± 0.23	0.836 ± 0.209	0.802 ± 0.055	0.730 ± 0.024	0.840 ± 0.211	0.973 ± 0.139
	TIMCO	0.770 ± 0.133a	0.734 ± 0.091a	1.10 ± 0.20ab	0.890 ± 0.224a	1.62 ± 0.12b	0.570 ± 0.150a	0.737 ± 0.093a
	MAGENTA	0.587 ± 0.062a	1.02 ± 0.07ab	1.18 ± 0.16b	1.37 ± 0.15b	− −	− −	− −
	ARRA15	1.06 ± 0.09	1.07 ± 0.14	1.05 ± 0.14	0.930 ± 0.200	− −	− −	− −
Methyl isobutyl ketone	CRIMSON	12.4 ± 0.5	12.2 ± 0.2	10.9 ± 0.1	10.9 ± 0.1	10.6 ± 0.2	10.4 ± 1.1	11.9 ± 2.1
	KRISSY	11.9 ± 0.4b	10.7 ± 0.3ab	10.7 ± 0.0ab	10.6 ± 0.0ab	11.9 ± 0.6b	10.3 ± 0.3a	11.8 ± 0.5b
	TIMCO	10.9 ± 0.7	11.9 ± 0.7	10.7 ± 0.5	11.2 ± 0.3	12.3 ± 0.5	11.5 ± 0.3	13.2 ± 1.3
	MAGENTA	11.9 ± 0.6b	9.88 ± 0.20ab	9.64 ± 0.66a	10.1 ± 0.4ab	− −	− −	− −
	ARRA15	10.6 ± 0.7	10.4 ± 0.3	10.9 ± 0.3	9.91 ± 0.71	− −	− −	− −
4-methyl-2-hexanone	CRIMSON	5.06 ± 0.17	4.95 ± 0.10	4.66 ± 0.30	4.40 ± 0.29	4.87 ± 0.25	4.80 ± 0.13	4.55 ± 0.02
	KRISSY	4.85 ± 0.16	4.71 ± 0.34	4.35 ± 0.56	4.49 ± 0.17	4.68 ± 0.01	4.66 ± 0.55	4.76 ± 0.05
	TIMCO	4.42 ± 0.02	4.64 ± 0.13	4.58 ± 0.47	4.52 ± 0.03	5.03 ± 0.62	4.71 ± 0.00	5.57 ± 0.51
	MAGENTA	4.51 ± 0.08	4.86 ± 0.27	4.47 ± 0.18	4.27 ± 0.28	− −	− −	− −
	ARRA15	4.61 ± 0.31	4.31 ± 0.44	4.70 ± 0.06	4.83 ± 0.19	− −	− −	− −
Isovalerone	CRIMSON	40.2 ± 5.6	40.6 ± 0.7	40.0 ± 1.5	40.0 ± 2.0	41.6 ± 3.3	39.6 ± 3.2	41.6 ± 5.7

	KRISSY	40.0 ± 2.6bbc	40.4 ± 1.2bc	38.7 ± 2.8bc	40.5 ± 0.9bc	36.3 ± 0.4ab	43.9 ± 3.0c	30.2 ± 0.6a
	TIMCO	36.3 ± 5.8	38.7 ± 1.4	37.1 ± 2.5	34.4 ± 5.8	38.7 ± 0.6	33.1 ± 0.9	37.5 ± 1.8
	MAGENTA	41.2 ± 0.2	40.8 ± 2.3	44.4 ± 0.2	36.1 ± 6.8	− −	− −	− −
	ARRA15	39.2 ± 3.2	35.5 ± 4.7	39.2 ± 0.7	41.1 ± 4.1	− −	− −	− −
Pentanoic acid	CRIMSON	2.08 ± 0.57a	6.42 ± 0.08ab	7.94 ± 0.37b	8.93 ± 0.08b	8.84 ± 1.43b	15.0 ± 0.1c	16.2 ± 2.9c
	KRISSY	2.54 ± 0.40a	5.26 ± 1.12ab	5.90 ± 1.04b	7.72 ± 1.25b	7.17 ± 0.24b	7.81 ± 0.46b	5.76 ± 0.25b
	TIMCO	2.78 ± 0.61a	3.19 ± 0.39a	5.70 ± 0.49ab	10.1 ± 2.5b	15.2 ± 1.6c	4.80 ± 0.04a	5.00 ± 1.27a
	MAGENTA	2.78 ± 0.11a	6.33 ± 1.15b	7.87 ± 1.13b	8.56 ± 0.06b	− −	− −	− −
	ARRA15	3.39 ± 0.98a	5.50 ± 0.09a	11.2 ± 0.9ab	19.0 ± 6.0b	− −	− −	− −
